# Iatrogenic Twiddler's Syndrome: Case Report and Proposed Experimental Model

**Published:** 2010-12-26

**Authors:** Francisco Femenia, Claudio Florentino, Martin Arrieta, Mauricio Arce

**Affiliations:** Unidad de Arritmias, Departamento de Cardiología, Hospital Español de Mendoza, Argentina

**Keywords:** Twiddler's Syndrome, pacemaker, complication, lead dislodgement

## Introduction

First described in 1968, pacemaker Twiddler's syndrome (TS) refers to permanent malfunction of a pacemaker or implantable cardioverter defibrillator, by dislodgement or fracture of the leads, due to conscious or unconscious manipulation of the pulse generator, causing rotation around its long axis [1-6].

## Case report

**Figure 1:**
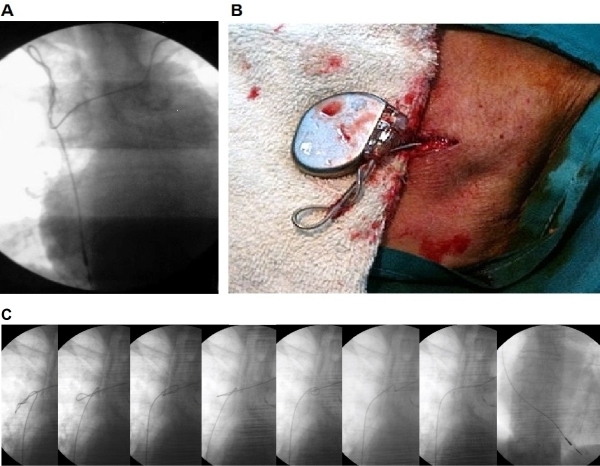
A: Antero-Posterior fluoroscopic view:  displacement of the electrode, corkscrew retraction about its longitudinal axis and migration to the right subclavian vein. B: Surgical revision of the pocket and lead: there is excessive twisting on the proximal portion of the lead. C: Antero-Posterior fluoroscopic view: From left to right lead reposition sequence, using a guide wire of 0.015". Gentle movements pulling back the lead were applied until complete reposition in the RV apex

A 74-year old woman, with history of systemic hypertension, dyslipemia, obesity and sick sinus syndrome (tachy-brady syndrome) underwent single chamber pacemaker implantation (Sensia SR, Capsure 5054 lead, Medtronic Inc, Minneapolis, MN, USA) via left subclavian vein puncture. The parameters at the implant were R- wave 12 mV, pacing threshold 0,4 V at 0,5ms, impedance 1200 Ω.  During the procedure, with the purpose of inserting the pulse generator in the pocket, some rotation exerted on the entire device (pulse generator and lead) was done to achieve a correct position. The patient-was discharged within 24 hours, without radiographic evidence of lead dislodgement. Parameters tested at discharge were R-wave 14 mV, pacing threshold 0,4 V at 0,5 ms, impedance 1200 Ω. Seven-days later, regular pacemaker check up was performed. Device interrogation demonstrated complete lack of sensing and pacing with normal lead impedance values. On closer interrogation, the patient admitted moving her arms energetically, but without applying any movements directly on the pulse generator. Fluoroscopy was indicated to determine the course of the lead. The ventricular lead was dislodged and lead retraction toward the right subclavian vein and twisted along its longitudinal axis was observed (Figure 1A). No clear evidence of pulse generator displacement was observed. Surgical revision was recommended. After opening the pocket, we extracted the pulse generator observing a complete twist of the proximal portion of the lead. (Figure 1B) The lead was fixed to the bottom of the pacemaker using the suture sleeve as usual. After opening the pocket, the suture lead was still present, however; not appropriately tied down. This may contribute to transmit the torsion from the pocket to the end of the lead.

Upon disconnection of the pulse generator, the tension was suddenly released, leading to complete resolution of the proximal twist. Using of a guide wire (0.015 ") advanced through the lumen of the lead, and pulling with gentle movements, a proper placement of the lead in the right ventricular apex, was achieved (Figure 1C). Parameters were in the normal range again. Pulse generator was inserted into the pocket and anchored to the bottom of the pocket. At the time of writing this report there were no complications during the follow-up.

## Discussion

**Figure 2:**
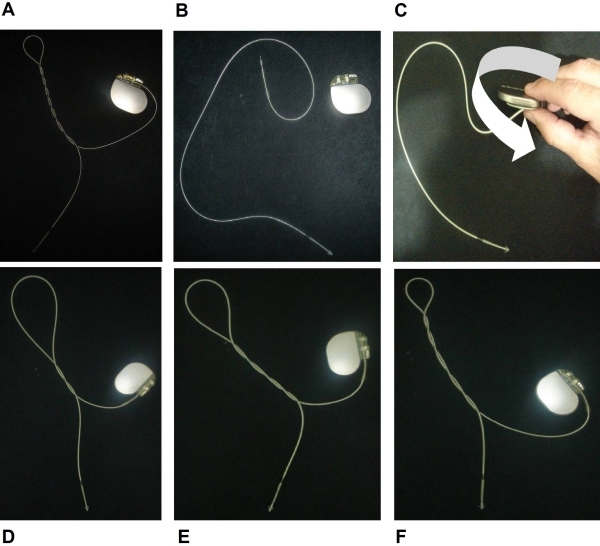
Experimental Model. A: twisting the lead around longitudinal axis (corkscrew) on one end while the other is fixed results in this unique lead appearance; B: to simulate the real case we use a similar pulse generator and lead; C: maintaining the electrode tip fixed, the generator is rotated clockwise and counterclockwise. D: twisting appearance after two laps; E: twisting appearance after four laps; F: twisting appearance after six laps.

Lead dislodgement is a clinically relevant and possibly dangerous complication. The incidence of early dislodgements (within the first 6 weeks of implantation) is ~3% [7-9]. Some mechanisms of pacemaker lead dislodgement involve retraction of the lead toward the generator. This includes the TS, which refers to lead dislodgement due to conscious or unconscious manipulation of the pulse generator causing the rotation of the lead around its long axis. In most TS cases, the reason remains unknown, but some predisposing factors have been identified (obesity, excessive movements of the upper limbs, active manipulation of the generator and large size pockets) [2,4,5,10-12]. As a consequence of the dislodgement (or fracture lead) sudden changes in impedance along with failure of capture (and/or sensing) may occur with potential fatal consequences. If risk factors for TS are detected during the implant, some preventive maneuvers like anchoring the pulse generator to the bottom of the pocket or sub-pectoral implants have been proposed [2,12,13]. TS is easily diagnosed with a chest X-ray and it should be part of the differential, when early lead dislodgement occurs. The vast majority of the cases have been reported in women, obese, with large pockets, facilitating the rotation of the pulse generator within the pocket. In our case, there was no displacement of the generator on its long axis, leading us to speculate that the dislodgement of the lead occurred due to torsion movements of the pulse generator during the implant, in our intention to place it within the pocket (iatrogenic TS). In order to explain what happened to our patient, we developed an experimental model to rigorously speculate the possible mechanisms involved in this case. To the best of our knowledge, this is the first experimental model to explain TS (Figure 2).

### Experimental model

**Figure 3:**
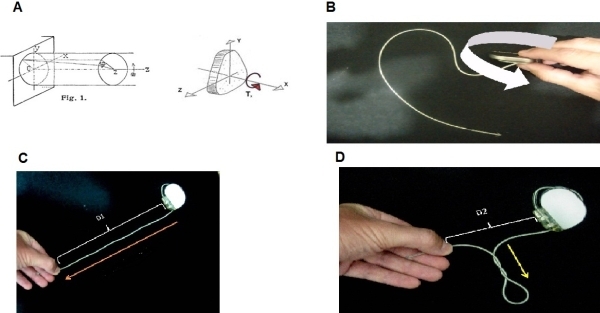
Representation of the physical phenomenon (see text)

The term torsion refers to the helicoidal deformation that a body may suffer when parallel forces are simultaneously applied with equal magnitude but opposite direction. This model is perfectly applicable to our experiment (implanted intra-cavitary catheter), were the predominant dimension is the length. The following figure represents this phenomenon (Figure 3). A cylindrical object is fixed at one end, and torque is applied to the other end (Figure 3-A). The twist is geometrically characterized when any curve parallel to the axis of the piece, is no longer in the plane initially constituted by the two curves. Instead a parallel curve to the axis is twisted around it. General studies of torsion are complicated and there are several simpler formulas for practical cases (warped pure torsion, Saint-Venant pure torsion, straight or twisting Coulomb theory). The rigorous solution of the problem, for any of the mentioned above can only be obtained by applying the theory of elasticity, which is beyond the scope of this discussion [14]. In our case, the lead is fixed in one end and the other is connected to a pacemaker pulse generator. The lead is exposed to several torque movements (twisting of the pulse generator in any direction: clockwise or counterclockwise) (Figure 3-B). These movements applied to the lead, generates a helicoidal deformation along the longitudinal axis, as explained above. It is important to note that due to the elastic characteristics of the leads and the force applied to them, it never gets outside the elastic deformation range. This means that if the applied forces are removed from the lead, it will return to its original point of equilibrium. On the other hand, the energy delivered to the lead by applying torque (twisting the pulse generator) is stored (in the lead) by the elastic properties of the lead (in the same way that a spring does). If traction force is applied, the lead will be stretched out and at this point the theory of straight torsion (also known as Coulomb) could be applied [14] (Figure 3-C). However, if the tractions force decreases, for example getting both ends closer, perpendicular deviations to the longitudinal axis could be detected (explained in the theory of Non-straight torsion). These deviation explain the effect of coiling the lead perpendicular to the longitudinal axis [14], resulting in the phenomenon shown in Figure 3-D.

## Conclusions

Iatrogenic TS is a rare complication of pacemaker implants. In pacemaker dependent patients it may result in a dramatic situation. Avoiding excessive rotations of the pulse generator to insert it in the pocket may prevent facilitating iatrogenic TS.
